# BNT162b2 vaccine considerations for immunocompromised individuals: A global perspective

**DOI:** 10.1016/j.amsu.2022.103796

**Published:** 2022-05-17

**Authors:** Hafsa Nazir Jatoi, Samina Abbas, Muhammad Saif Abbasi, Misha Asif Tauni, Shamas Ghazanfar, Mohammad Daniyal Zafar Malick, Muhammad Faiq Umar, Muhammad Junaid Tahir, Muhammad Sohaib Asghar, Ali Ahmed

**Affiliations:** a Dow Medical College, Karachi, Pakistan; b Mayo Hospital, Lahore, Pakistan; c Lahore General Hospital, Lahore, 54000, Pakistan; d Dow University of Health Sciences–Ojha Campus, Karachi, Pakistan; e School of Pharmacy, Monash University, Jalan Lagoon Selatan, Bandar Sunway, 47500, Subang Jaya, Selangor, Malaysia

**Keywords:** COVID-19, SARS-CoV-2, Infection, Vaccine hesitancy, Vaccination, Comorbidity

## Abstract

With the emergence of COVID-19 vaccines, individuals with comorbidities and immunosuppression require particular attention and should be prioritized for vaccination. However, the majority of vaccine clinical trials excluded people with comorbidities, resulting in a lack of data regarding vaccine efficacy in this demographic. Along with more inclusivity in clinical trials, reaching a definitive conclusion regarding vaccine efficacy in these patients is also crucial. In our review, we highlight the BNT162b2 vaccine safety and efficacy based on the limited number of clinical trials which included this demographic. We also provide vaccine considerations for individuals with cancer, autoimmune diseases, HIV, obesity, diabetes, organ transplant recipients and those undergoing maintenance haemodialysis to help them govern their decision regarding vaccine administration. In conclusion, further studies are required to alleviate any insecurities in patients with comorbidities regarding vaccination and it is recommended that patients are monitored post-vaccination to make sure sufficient immunity is achieved.

## Highlights

• With the emergence of COVID-19 vaccines, individuals with comorbidities and immunosuppression should be prioritized for vaccination.
• In our review, we highlight the BNT162b2 vaccine safety and efficacy in immunocompromised individuals.
• Further studies are required to alleviate any insecurities in patients with comorbidities regarding vaccination.

## 1 Introduction

Severe acute respiratory syndrome coronavirus-2 **(**SARS-CoV-2) has affected global health care and economic systems. It is known to affect people of all age groups and genders; however, the duration of hospitalization and consequences in the coronavirus disease 2019 (COVID-19) patients were greater in people of older age as well as people of any age with underlying illnesses such as chronic obstructive pulmonary disease (COPD), diabetes, and hypertension [1]. The risk of infection of COVID-19 was found to be 4-fold higher in patients with COPD compared to those without [2]. People with comorbidities had a rapid and severe progression of the disease, resulting in death related to COVID-19 (1). To reduce the number of infections and deaths in this population, they must be prioritized for vaccination.

The rapid development and administration of vaccines on a wide scale continues presently. However, most trials of COVID-19 vaccines did not include immunocompromised individuals, which has led to limitations of data available regarding vaccine safety in this population [3]. Hence, there are differing views regarding the vaccine's efficacy amongst such patients. A study involving 996,500 individuals reported increased hesitancy in 13.4% (731/5459) patients with cancer, 19.4% (964/4947) patients with autoimmune diseases, and 17.8% (1344/7544) patients with chronic lung diseases [3].

Whether or not these concerns, regarding vaccine's safety and efficacy amongst individuals at risk, are justified can be seen from the results of an observational study in the United Kingdom (UK), which reports that there was a 54% risk reduction of infection due to COVID-19, in patients with comorbidities after being administered the Pfizer-BioNTech (BNT162b2) and the Oxford-AstraZeneca (ChAdOx1 nCoV-19) COVID-19 vaccines [4].

**Fig. 1:**
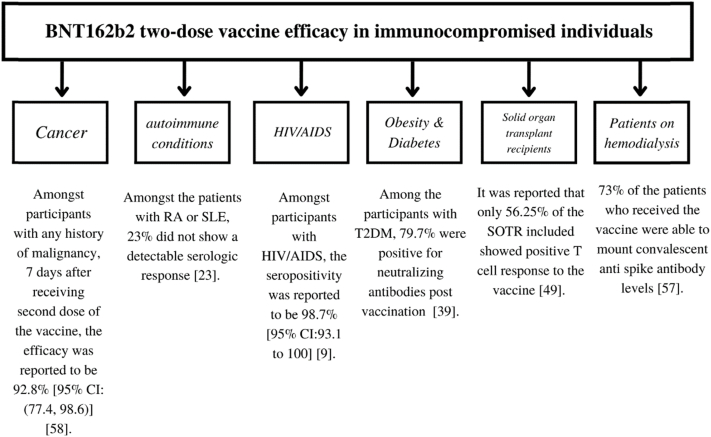
Effectiveness of BNT162b2 after second dose in immunocompromised individuals based on published literature [58].

The vaccine hesitancy widely stems from the lack of knowledge and clinical trials for the COVID-19 vaccine which included individuals at risk. To increase vaccine acceptance, these vaccine safety concerns must be addressed, and, in this review, we explore the considerations of BNT162b2 in people at high risk of contracting the COVID-19 infection due to immune compromise or comorbidities, based on clinical trials and published research (Fig. 1).

## 2 Efficacy and safety of BNT162b2 vaccine based on trials

Although a significant number of COVID-19 vaccines have been prepared, only those with high safety and efficacy rates would be suitable for patients with comorbidities and the elderly [5]. Placebo-controlled, observer-blinded clinical trials of the BNT162b2 vaccine included 43,448 participants, developed by Pfizer and BioNTech, involved patients aged 16 or above and patients with comorbidities [6]. The trials showed a good vaccine safety profile with an efficacy of over 95% [CI: 90.3, 97.6] in adults aged 65 years or above and no adverse effects were reported [7,8]. In another recent clinical trial of the BNT162b2 vaccine amongst five groups of immunocompromised individuals, it was found that the vaccine was effective in 72.2% of immunocompromised individuals compared to 100% of healthy controls [9]. Although vaccine effectiveness of BNT162b2 in patients with comorbidities was slightly lower in comparison to healthy individuals, it is sufficient to develop significant immunity in these patients [10]. It is further suggested that additional doses of vaccine be set aside for immunocompromised patients to improve their immunity [9]. Contradictory to this trial's results, in another trial, out of 37,706 participants, 24% constituted people with comorbidities which included diabetes mellitus (8.4%), hypertension (24.5%), obesity (35.1%), and chronic lung disease (7.8%) [8,11]. Vaccine effectiveness was found to be similar in patients with hypertension (95.4%; 95% CI, 82.6 to 99.5), diabetes mellitus (94.7%; 95% CI, 66.8 to 99.9), and chronic lung disease (93.0%; 95% CI, 54.1 to 99.8) as compared to healthy individuals. A total number of six deaths occurred, two in the vaccine group and four in the placebo group. However, these deaths were not associated with vaccine administration [11]. These differences in trial results could be due to the sample size or age of the involved individuals. Hence, there is further need for trials to include all age groups for each comorbidity in order to highlight the vaccine efficacy based purely on comorbidity (age and sex-adjusted).

## 3 BNT162b2 vaccination in at-risk individuals

### 3.1 Cancer patients

Cancer patients are at an increased risk of a severe form of COVID-19 infection, poor prognosis, and death [12,13]. These studies suggest that cancer patients must be prioritized for COVID-19 vaccination and patients with an advanced stage of cancer are recommended to be vaccinated first in case their systemic treatment allows for a one-month delay [14]. However, data regarding the involvement of patients with cancer or previous history of cancer in the clinical trials of the development of the COVID-19 vaccine remains insufficient because, in most of the clinical trials, patients with active cancer or those undergoing treatment such as chemotherapy, radiotherapy, or immunosuppressant were excluded from the trials [15]. This leads to increased vaccine hesitancy as mentioned previously [3]. The benefits of getting vaccinated far outweigh the risks for cancer patients, even if they develop some amount of protection, it will be beneficial in the long run because they have frequent hospital visits for treatment which exposes them to the virus more than rest of the population [16].

Due to limited data regarding the safety of the COVID-19 vaccine for cancer patients, extrapolating data from the past, and looking back during the time of administration of the influenza vaccine in cancer patients, it can be inferred that a significant amount of immunity developed against influenza. Hence, providing evidence that the development of sufficient immunity is not an obstacle in these patients [17] and, based on a study in Germany involving patients with breast cancer and gynaecological malignancies, it was found that cancer patients have a tolerance for the COVID-19 vaccination whilst taking their on-going cancer therapy, without any other significant side effects apart from those usually reported in the general population [18]. Of the 300 cancer patients involved in the study, 77.5% received the Pfizer vaccine. The vaccine had a significant safety report, and all local and systemic adverse events were found to be self-limiting, most lasting for less than 48 h after vaccination. Results suggested no adverse events due to the vaccine [18].

High efficacy vaccines such as BNT162b2 (efficacy greater than 94%) are hence, likely to generate a sufficient protective response in cancer patients [19]. However, setting a timeline for vaccine doses and intensive therapies is extremely essential for these patients. For example, the vaccine should be administered after an appropriate recovery time after surgery or high-intensity treatments (such as chemotherapy) [19]. Also, live vaccines are not recommended in cancer patients because of their weakened immunity [17].

### 3.2 Individuals with autoimmune conditions

Similar to cancer patients, based on research, individuals with autoimmune systemic diseases (ASD) are also more vulnerable to SARS-CoV-2 infection, a higher incidence of hospitalization and morbidity due to an impaired immune system, and ongoing immunosuppressive therapies [[20], [21], [22], [23]]. Moreover, an incompetent immune system due to ongoing immunosuppressive therapies may fail to show significant neutralizing antibody titres after vaccination, Ammitzbøll et al. reported that among the patients with Rheumatoid Arthritis (RA) and Systemic Lupus Erythematosus (SLE), 23% of the patients were unable to show a detectable serologic response after two doses of BNT162b2 vaccine [24]. Trials for BNT162b2 did not include individuals with underlying autoimmune conditions and those on immunosuppressive therapies hence the quality of immune response is not well understood in these potentially vulnerable populations [25].

The majority of patients suffering from autoimmune inflammatory rheumatoid diseases (AIIRD) do show an immune response after vaccination with BNT162b2 however, the S1/S2 IgG neutralizing antibodies measured 2–6 weeks after the second vaccine dose were found to be substantially lower in patients with AIIRD compared to the controls which raise concerns about long term protection [26]. Furthermore, glucocorticoids (GC) are an important constituent of AIIRD treatment and the seropositivity rate in individuals on GC therapy was 66% only, although the data available on the sole effect of GC on immune response post vaccination is limited [26]. Another study shows that the BNT162b2 vaccine triggers significant immune responses in most individuals (94%) suffering from immune-mediated inflammatory diseases (IMID) however, the percentage drops to 30% in patients on B-cell depleting agents [27].

Moyon et al. reported that SLE activity at the time of vaccination did not affect vaccine efficacy and there was no risk of increased SLE flares or vaccine side effects, although it should be noted that treatments received by individuals with active SLE may hamper the antibody response to vaccination, specifically drugs methotrexate and mycophenolate mofetil are associated with low baseline IgG levels and a reduced pool of naïve B cells [28]. Another study reported that 95% of the patients with systemic inflammatory diseases treated with rituximab did not produce a neutralizing response against variants of COVID-19 [29].

Since the start of vaccination drives there have been questions regarding the safety of vaccines, especially considering individuals with autoimmune conditions, a study from Japan reported that 10.2% (19 of 187) of individuals with inflammatory bowel disease (IBD) were hesitant towards vaccination while a study from Kuwait also reported increased vaccine hesitancy among people with IBD [30,31]. Studies do provide evidence that there are significant immune responses in individuals with multiple sclerosis and that there is no increased risk of relapse after vaccination but more research into other autoimmune conditions is required to tackle vaccine hesitancy [32].

### 3.3 HIV/AIDS patients

HIV or AIDs is classified as another independent risk factor for increased mortality due to COVID-19. Although it is suggested that immunocompromised individuals especially those with HIV be prioritized for the vaccine, due to the limited clinical data available for this demographic, there are still gaps in the knowledge regarding the efficacy of BNT162b2 in this cohort similar to those mentioned previously [33].

Efficacy of the vaccine in such patients is largely measured using CD4^+^ cell count, viral load, and disease stage [33]. According to a recently published prospective study including 143 HIV patients aged above 18 years and 261 healthy HCWs used as control, it was found that 18 days after the second dose of BNT162b2 vaccination, 98% (139/141) HIV patients had positive anti-receptor binding domain (RBD) IgG while 98.9% (258/261) HCWs had positive anti-RBD IgG 26 days after the second dose of BNT162b2 vaccine [34]. Regardless of this discrepancy in the results, the population under study had similar neutralizing activity. No AIDS-related adverse event was reported in this sample population. Although not related to any clinical signs or symptoms, HIV load was found to have increased in 2% (3/143) patients while a decrease was seen in CD4^+^ T-cell count, which suggests that there is a need for further monitoring in upcoming trials. It is also found that spike levels of IgG antibodies are lower in HIV patients than in healthy individuals [35,36]. Similarly, according to an observational study testing the efficacy of mRNA-1273 and BNT162b2 vaccines in HIV patients based on the humoral response, it was found that mRNA-1273 had 5.47 higher odds of surrogate virus neutralization test (sVNT) response (95% CI) [37]. 28% of BNT162b2 recipients and 12% mRNA-1273 recipients showed non-responsive sVNT. Of those showing IgG non-response, 12 received BNT162b2 while none received mRNA-1273 [37]. These results suggest that the BNT162b2 mRNA vaccine although slightly less effective in HIV patients is immunogenic in those with a retained immunity [34]. Hence, targeted vaccine strategies including post-vaccine serology and a quicker process for further doses of the vaccine should be considered in this cohort [36].

### 3.4 Obese and diabetic individuals

Studies have shown that obese people with type 2 Diabetes Mellitus (T2DM) may have impaired innate/adaptive immunity, which is a result of the production of several pro-inflammatory cytokines (INF-γ, TNF-α, IL-1) due to which they may be more susceptible to a severe COVID-19 infection [38,39]. Considering these risks, people with T2DM have been prioritized for vaccination although the results have not been too promising, Ali et al. reported that after administration of the BNT162b2 vaccine there are significantly lower antibody titres in people with type 2 diabetes compared with non-diabetics, and these results are further backed by a study from Japan which shows that Haemoglobin A1c level higher than 6.5 significantly suppresses the antibody responses to the BNT162b2 vaccine [39,40].

Similar to T2DM, obesity and excess visceral fat have also been identified as major risk factors to develop severe COVID-19 complications, especially in the young [41]. Chronic low-grade inflammation present in obese people may weaken immune responses including those mediated by T cells hence, immune response post vaccination may be impaired [42]. A cohort study on healthcare workers reported that antibody titres in obese individuals (body mass index (BMI) of 30 or greater) showed significantly lower increases in neutralizing antibody titres after administration of the second dose of BNT162b2 vaccine compared with individuals with a BMI of less than 25 [43]. Another study assessing the correlation between abdominal obesity (AO) and response to mRNA vaccines reported that after two doses of BNT162b2 vaccine lower antibody peak was found among infection-naïve individuals with AO compared to individuals without AO [44]. Contradictory to these findings, some studies have also found comparable vaccine efficacies in individuals with obesity and those without [45,46].

### 3.5 Solid organ transplant recipients

Solid Organ Transplant Recipients (SOTR) is another group of individuals who are at a higher risk of morbidity and mortality by the SARS-CoV-2 virus. Kates et al. reported that the mortality in hospitalized SOTR who contracted COVID-19 was 20.5% [47]. This is not surprising at all due to the reduced antibody responses in this cohort. Multiple studies [48] have been conducted to assess the effectiveness of the BNT162b2 vaccine in SOTR, only to find out that the BNT162b2 vaccine has no significant effect on SOTR. Miele et al. reported that only 56.25% of the SOTR included showed a significant T-cell response to the vaccine [49]. A similar study conducted on Liver Transplant Recipients (LTR) in Germany also reported similar results, where the authors reported a significant difference in the level of IgG response between vaccinated LTR, LTR post-COVID infection, and the control [50]. Vaccinated LTR showed no specific SARS-CoV-2 IgG even after both doses of the vaccine, as opposed to LTR who had been previously infected with COVID-19 demonstrating elevated titres of the anti-SARS-CoV-2 IgG in 85% of the patients approximately three months post-diagnosis [50]. An interesting observation is the use of the same immunosuppressive protocol for both arms of the study, although this was not a randomized controlled trial, except for mycophenolate mofetil being discontinued in patients diagnosed with COVID-19. Havlin and colleagues hypothesized that a differing and long-lasting response in response to infection in contrast to the vaccine would be implicated in this finding [50]. Another intriguing finding in this study was the evidence of a T-cell response to vaccination in a minority of patients (4/12) even though the authors failed to detect IgG in the serum. Furthermore, the three vaccinated individuals who contracted the infection did show very mild symptoms, hinting at the notion of the vaccine working in ways yet undiscovered.

Korth et al. found similar results when comparing renal transplant recipients to healthy healthcare workers, all of whom received the BNT162b2 vaccine [51]. The study found a vast disparity in the responses shown by SOTR and healthy control group with only 22% (5/23) of the samples from transplant recipients showing significant IgG titres as opposed to all the samples from the healthy individuals exhibiting consequential IgG levels. This can be attributed to the impaired immunity amongst SOTR [51]. Boyarsky et al. found only 17% (76/436) of the vaccinated SOTR possessed significant antibody titres. Another fascinating finding in this study was that individuals who received mRNA-1273 (69%) had a higher likelihood of developing significant antibodies to the disease as compared to the recipients of BNT162b2 (31%) (P = 0.003) [52].

All of this points to the fact that despite vaccination, SOTR is more susceptible to the COVID-19 infection and implores the medical community to find a regiment that is more suited to these individuals. A potentially viable solution could be the mix and match of multiple vaccines to elicit a substantial immune response and prevent unwanted side effects.

### 3.6 Patients undergoing maintenance haemodialysis

Patients undergoing haemodialysis have a considerably greater risk of being infected by the COVID-19 virus, and significantly greater morbidity and mortality than the average population [53,54]. Therefore, multiple studies were undertaken to find the efficacy of the BNT162b2 vaccine in these patients. Hasse et al. studied a cohort to find the humoral immunogenicity in the population vaccinated by the BNT162b2 vaccine as compared to the AstraZeneca ChAdOx1-S-nCoV-19 vaccine [55]. In their prospective study, Hasse and colleagues elucidated a heterologous effect of both vaccines to be much greater than the humoral response by either one of the vaccines alone [55].

Studies also discovered that vaccine efficacy deteriorates over time due to haemodialysis. Hsu and colleagues found that the anti-spike IgG titre values dropped substantially in six months, such that they were less than the effective titre values [56]. The study reported that with the BNT162b2 vaccine the values went from ≥ 20 to 1.99, during a six-month-period post-vaccination [56]. Another interesting finding of the study was that with the mRNA-1273 (Moderna) vaccine, the decline in titre values was considerably lower (≥20 to 7.99) during the same period [56]. Angel-Korman et al. uncovered the rate of decrease of the antibody levels in patients with haemodialysis to be significantly greater than in the control group [57]. The study also disclosed that initial levels of serum antibody in the patients vaccinated with the mRNA-1273 was much greater than the ones vaccinated with the BNT162b2 vaccine which could explain the titre values being greater six months post-vaccine. Hence, it is very critical to include these informations to educate the scientists and then educate the population in general. This review can help to create public health policies, improving the knowledge of the vaccines and the disease-prevented.

## 4 Conclusions

With the currently available data, it is suggested that despite the likelihood of diminished immune response in this population comorbid patients should be prioritized for vaccination. However, assuming that this population would eventually recognize the benefits of the vaccine while ignoring the risks is invalid. It is recommended that the government with the cooperation of health care departments initiates projects aimed at increasing awareness regarding the high benefit to risk ratio of COVID-19 vaccines and have dialogues that aim to discuss the effects of the vaccine on the underlying illnesses of comorbid individuals. Effective measures for vaccination carried out promptly in these patients will greatly improve the quality of care provided to this population during these difficult times, allowing for their improved and sustained health. Prompt clinical application of this knowledge could potentially result in a lower number of COVID-19 cases and reduced financial, hospital, and psychological pressure on both healthcare workers and society. Finally, due to the apparent lack of knowledge and data regarding the impact of the COVID-19 vaccine trials in distinct populations, along with prompt vaccine strategies, there is a need for increased research and inclusivity of people with comorbidities in clinical trials for the COVID-19 vaccine.

## Sources of funding

None.

## Ethical approval

Not required.

## Consent

Not required.

## Author contribution

H.N.J and S.A conceived the idea; H.N.J, S.A, M.S.A, M.A.T, S.G, M.D.Z.M, M.F.U, M.J.T, and A.A did write up of the manuscript; and finally, A.A, M.S.A, and M.J.T reviewed and revised the manuscript for intellectual content critically. All authors approved the final version of the manuscript.

## Registration of research studies

1. Name of the registry: Not required.
2. Unique Identifying number or registration ID: N/A
3. Hyperlink to your specific registration (must be publicly accessible and will be checked):

## Guarantor

Muhammad Sohaib Asghar and Muhammad Junaid Tahir.

## Provenance and peer review

Externally peer reviewed, not commissioned.

## Declaration of competing interest

None.
